# Case Report: α1-antitrypsin deficiency causing persistent pleural effusion and multilobar alveolar emphysema in a young dog

**DOI:** 10.3389/fvets.2025.1678702

**Published:** 2025-10-28

**Authors:** Pavlos G. Doulidis, Carolina Frizzo-Ramos, Christof A. Bertram, Olivia M. Grünzweil, Sibylle M. Kneissl, Brigitte Degasperi, Reinhard A. Hirt, Iwan A. Burgener, Nicole Luckschander-Zeller

**Affiliations:** ^1^Clinical Unit of Internal Medicine Small Animals, Department for Companion Animals and Horses, University of Veterinary Medicine, Vienna, Austria; ^2^Clinical Unit of Anesthesiology and Perioperative Intensive Care, Department for Companion Animals and Horses, University of Veterinary Medicine, Vienna, Austria; ^3^Institute of Pathology, University of Veterinary Medicine, Vienna, Austria; ^4^Diagnostic Imaging, Department for Companion Animals and Horses, University of Veterinary Medicine, Vienna, Austria; ^5^Small Animal Surgery, Department of Companion Animals and Horses, University of Veterinary Medicine, Vienna, Austria

**Keywords:** emphysema, pulmonary, pleural effusion, canine, alpha-1 antitrypsin (AAT), histopathology

## Abstract

Alpha-1 antitrypsin deficiency (A1ATD) is a rare genetic condition in both humans and animals, caused by mutations in the SERPINA1 gene that lead to reduced or absent production of alpha-1 antitrypsin (A1AT). This case report describes a 3-year-old male dog presenting with persistent pleural effusion, chronic nonproductive cough, and respiratory distress. Despite an extensive diagnostic evaluation that included computed tomography (CT), a definitive diagnosis of A1ATD was only reached after a histopathological examination of lung tissue, which revealed acinar emphysema characterized by the destruction of alveolar walls. Serum A1AT levels were undetectable, confirming the diagnosis. The absence of liver involvement aligned with the lung-predominant phenotype described in human A1ATD. This is the first reported case of A1ATD-associated emphysema and pleural effusion in a dog, emphasizing the need for further research into its pathophysiology, diagnosis, and management in canine patients.

## Introduction

Alpha-1 antitrypsin (A1AT) is a serine protease inhibitor that blocks neutrophil elastase and helps maintain protease/antiprotease balance in the lung (1). A1AT also demonstrates antioxidative and anti-inflammatory properties (2). It is primarily produced in the liver by hepatocytes, but also by alveolar monocytes and macrophages (3, 4). Genetic mutations in the SERPINA1 gene can result in reduced or no production of A1AT from birth or early life (5). Alpha-1 antitrypsin deficiency (A1ATD) is an underdiagnosed autosomal genetic disorder in humans that leads to liver disease and emphysema, often presenting with chronic obstructive pulmonary disease (COPD) and small airway dysfunction (6, 7). Reports of A1ATD in dogs are very rare, and comprehensive studies are lacking; however, the limited information available suggests that genetic, diagnostic, and potential therapeutic pathways may be similar to those in humans (8–10). To the best of our knowledge, this is the first case report describing a dog with A1ATD presenting with persistent pleural effusion and pulmonary emphysema.

### Case description

**Figure 1:**
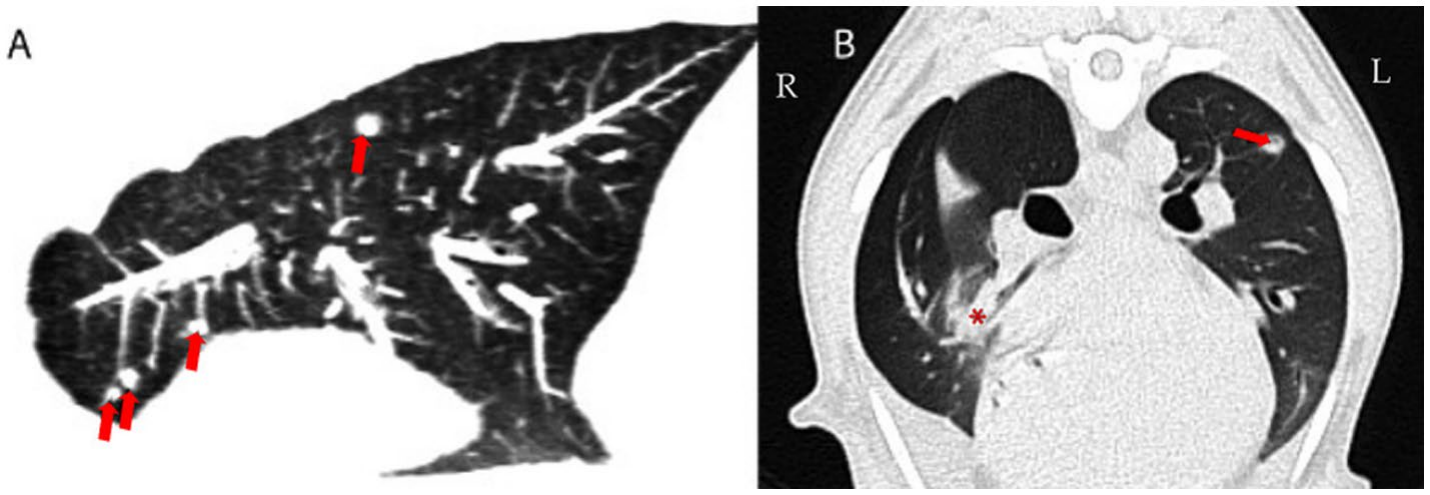
First CT study: Sagittal reconstruction of the left lung (maximum intensity projection, 3 mm thick section) **(A)** and transverse CT image at the level of the right middle lung lobe (averaged intensity projection; 0,7 mm thin section; WL -600, WW 1200) **(B)**. Round shadows in the left lung lobes (arrows) and multifocal consolidation with air bronchograms (demonstrated in the right middle lung lobe; asterisk) and pleural effusion were the major findings. Attenuation of non-consolidated lung parenchyma: -850 to -800 Hounsfield Units (HU) (reference range of normal lung tissue: -846 to -713 HU (34).

**Figure 2:**
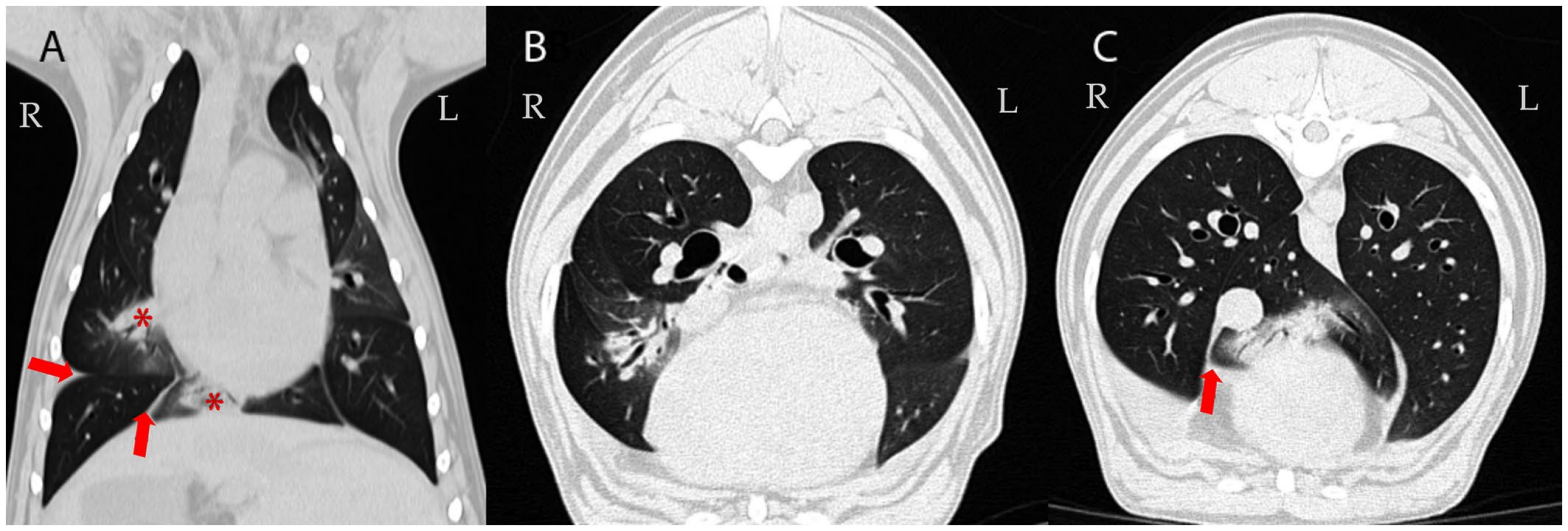
Follow-up CT examination 78 days later: Dorsal **(A)** and transverse CT images of the lung **(B,C)**; averaged intensity projection; 0,6 mm thin section; WL -600, WW 1200. Multifocal airway-associated consolidation (demonstrated in the right middle and accessory lung lobes; asterisk in **A**) and pleural effusion persisted (arrows). Attenuation of non-consolidated lung parenchyma: -850 to -800 Hounsfield Units (HU) (reference range of normal lung tissue: -846 to -713 HU (34).

A 3-year-old male intact mixed-breed dog was referred to the Small Animal Internal Medicine Unit of our university for further investigation of transudative pleural effusion, chronic non-productive cough, chronic expiratory respiratory distress, lethargy, reduced appetite, and weight loss. The dog lived in an urban area with no known exposure to smoke, toxic substances, or heavy metals. The referring veterinarian had performed routine blood work, including a CBC, serum biochemistry, and C-reactive protein (CRP) testing, all of which were within normal limits. Thoracic radiographs revealed the presence of pleural effusion. Thoracic computed tomography (CT) revealed moderate pleural effusion and multiple small, round nodules in the left lung lobes, measuring 1–4 mm in diameter (Figure 1). Differential diagnoses included inflammatory lesions, granulomas, small areas of atelectasis, and neoplasia. Effusion analysis performed by the referring veterinarian, including total protein, cytology, LDH, and cell count, classified the pleural effusion as a transudate. No etiological cause for the transudate in the pleural space or persistent cough could be identified by the referring veterinarian. Prior to referral, the dog was treated with several medications, including antibiotics (amoxicillin with clavulanic acid, enrofloxacin, and doxycycline), furosemide, meloxicam, antihistamines, and a short course of dexamethasone at an anti-inflammatory dosage, without clinical improvement. However, detailed dosage regimens were not available. Upon first clinical examination, the dog demonstrated mild expiratory respiratory distress, a body condition score of 4/9, and increased lung sounds cranially on both sides, while the rest of the clinical examination was unremarkable. Upon admission, blood tests, including CBC and serum biochemistry, were repeated. The hematocrit was at the lower limit (37%; RI 37–55%), while total protein and albumin were slightly decreased (4.24 g/dL, [RI 6.00–7.50 g/dL] and 2.29 g/dL [RI 2.58–4.73 g/dL], respectively). Blood urea nitrogen was increased (48.5 mg/dL; RI 20–40 mg/dL). CRP was within normal range (0.8 mg/dL; RI < 35 mg/dL) and remained low throughout all future rechecks. After a positive fecal occult blood test, the above findings were attributed to gastrointestinal (GI) bleeding, probably due to prior treatment with meloxicam and dexamethasone. With treatment with omeprazole (1 mg/kg IV q12h), sucralfate (30 mg/kg PO q8h), and maropitant (1 mg/kg IV q24h), the GI bleeding ceased, and clinical parameters normalized within 5 days. Liver function testing (bile acids, ammonia, and coagulation panel) and serum osmolality (302 mOsm/kg; RI: 290–310 mOsm/kg) were within normal limits. Analysis of the pleural effusion confirmed a pure transudate, with a total protein of 0.5 g/dL and no inflammatory cells. Bacterial culture of the transudate was negative. Echocardiography revealed no abnormalities. Antigen enzyme-linked immunosorbent assay (ELISA) tests for *Dirofilaria immitis* and *Angiostrongylus vasorum* were negative. Fecal flotation and larval migration (Baermann–Wetzel test) for lungworms were also negative. Coagulation testing, including D-dimer and viscoelastic analysis, ruled out a thrombotic state. Serum antinuclear antibody levels were within normal limits, and serum protein electrophoresis was unremarkable. Furthermore, serologic testing for *Leishmania infantum*, *Ehrlichia canis*, and *Borrelia burgdorferi* yielded negative results (all below 1:20). Urinalysis was unremarkable, and the urine protein-to-creatinine ratio (UPC) was within normal range. Abdominal ultrasound showed no abnormalities except slight signs of gastroenteritis. A follow-up thoracic CT scan (Somatom X.cite VA30, Siemens Healthcare GmbH, 91,052, Germany), performed 78 days after the first one, was followed by bronchoscopy with bronchoalveolar lavage (BAL) and ultrasound-guided fine-needle aspiration (FNA) of the lung. On CT, the previously described lung nodules (round shadows) were no longer present; however, multifocal consolidation of the right lung lobes and pleural effusion were noted (Figure 2). Flexible fiberoptic bronchoscopy, along with cytologic evaluation of both the BAL fluid and the FNA from a consolidated area of the right lung, revealed predominantly alveolar macrophages with very few neutrophils and was considered unremarkable. Bacterial cultures and susceptibility testing detected very low concentrations of *Pasteurella multocida,* which was sensitive to amoxicillin. Polymerase chain reaction (PCR) testing for *Mycobacterium* spp.*, Toxoplasma gondii*, and mycology from both the pleural effusion and pulmonary FNA material yielded negative results. After exclusion of all the above mentioned potential causes, differential diagnoses included rare causes of interstitial lung disease, such as environmental and occupational exposures (e.g., asbestosis, silicosis, and extrinsic allergic alveolitis), pulmonary alveolar proteinosis, parasitic infections (*Pneumocystis carinii* and *Mesocestoides*), A1ATD, lipid pneumonia, systemic inflammatory diseases (e.g., lupus or polyarteritis nodosa), and, finally, idiopathic interstitial pneumonia.

**Figure 3:**
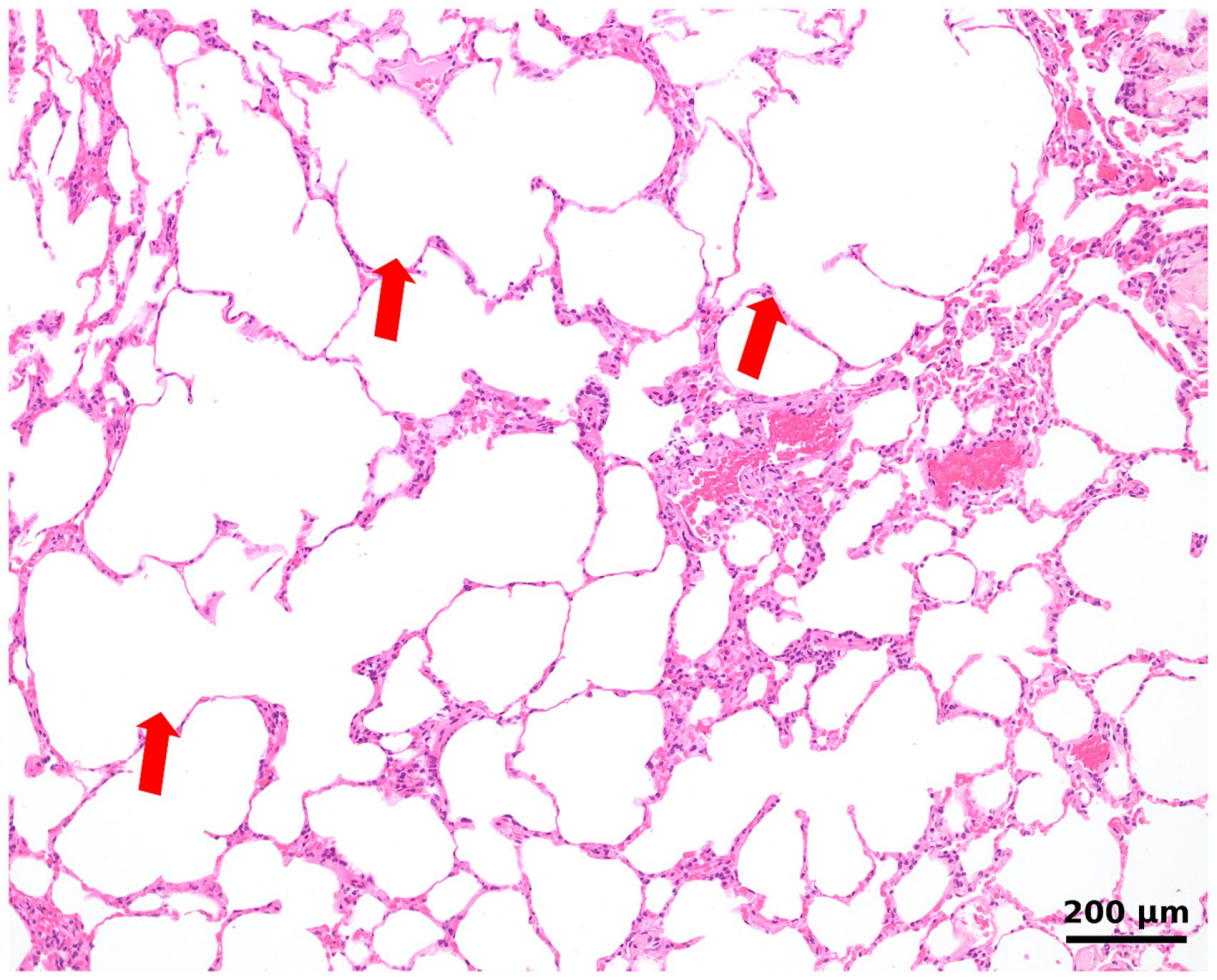
Histologic image of the lung shows largely expanded alveoli with the confluence of alveolar spaces, consistent with emphysema (arrows). The lung is devoid of inflammation.

As no etiological diagnosis could be established and the patient continued to experience recurrent pleural effusions despite multiple thoracocenteses, surgical biopsy and histopathology of the lung and pleura were performed. After right lateral thoracotomy, a total lobectomy of the right middle lobe and a biopsy of the parietal pleura were performed. A right lateral thoracotomy was selected because imaging demonstrated the most pronounced lesions in the right hemithorax, and the right middle lobe was both severely affected and surgically the most accessible. Grossly, the affected lobe exhibited regions of overexpansion with thin, fragile parenchyma interspersed with areas of collapse, consistent with emphysematous changes and atelectasis. The remaining lung lobes, pleura, pericardium, mediastinum, and regional lymph nodes appeared unremarkable on both inspection and palpation. Complete lobectomy was performed both to obtain sufficient tissue for histopathological examination and to remove the most diseased parenchyma, following standard surgical technique. For the management of the persistent pleural effusion, a 14F pleural port (PP102K, Norfolk Vet Products Inc., 60,076 Illinois, USA) was installed in the eighth right intercostal space. For histopathological examination of the dissected lung and pleura, routine tissue sections stained with hematoxylin and eosin were produced. Regions of largely expanded alveoli with confluence of alveolar spaces due to destruction of alveolar walls/septa (emphysema) (Figure 3) were intermixed with regions of almost complete alveolar collapse. Alveolar collapse was interpreted as compression atelectasis secondary to pleural effusion. Atelectatic regions were characterized by closely arranged alveolar walls interspersed by slit-like residual alveolar spaces. No inflammation was found within the pulmonary airways, interstitial space, visceral pleura, or vessels. Special stains for iron revealed few alveolar macrophages containing hemosiderin. No fungi (such as *Pneumocystis carinii*) or other infectious agents were detected using the periodic acid-Schiff (PAS) reaction.

In addition to histopathology, bacterial and mycological cultures of the tissues, and PCR for *Mycobacterium* spp., were performed and turned out to be negative. Based on histological findings, A1ATD was considered a possible differential diagnosis for the emphysema and the persistent pleural effusion. Quantitative measurement of serum A1AT was performed with a sandwich ELISA similar to what was previously described in the literature (11). In two consecutive measurements, the concentration of serum A1AT was unmeasurably low (<180 mg/L) compared to a surplus serum sample from a healthy dog, strongly suggesting a diagnosis of A1ATD. To further confirm the diagnosis, a second serum A1AT measurement was performed at a different laboratory using the immunoassay described by Heilmann et al. (12). The A1AT concentration was again low (13.6 mg/L, RI 732–1802 mg/L). The patient was discharged 3 days after surgery with supportive treatment and instructions to drain the effusion through the pleural port if persistent respiratory distress occurred, as no causal treatment for this condition in dogs has yet been described. During follow-up examinations at 2 weeks, 4 weeks, 2 months, 6 months, and 1 year, the cough had resolved, and the dog remained clinically healthy, aside from persistent pleural effusion. Drainage via the port was initially needed biweekly and later reduced to once a month.

## Discussion

The first cases of A1ATD in humans were described more than 50 years ago, and since then, various phenotypic variations with differing disease severity and prognosis have been reported (13, 14). Alpha-1 antitrypsin deficiency is a rare genetic disorder that can cause emphysema in humans and is commonly mistaken for asthma (5). The pathophysiology is attributed to the lack of A1AT function, which normally protects lung tissues, largely by neutrophil elastase, from proteolytic destruction (5). Chronic deficiency of A1AT can lead to severe emphysema and liver cirrhosis due to the lack of inhibition of neutrophil elastase (15, 16). To date, the only available treatment in humans is augmentation therapy, which is considered safe, although the effectiveness and benefit are controversial (17, 18). Augmentation therapy involves the intravenous administration of A1AT derived from pooled human plasma to increase serum levels and protect lung tissue from proteolytic damage. Augmentation therapy can lead to a slower progression of emphysema in patients with severe A1ATD by reducing the decline in lung function (19). Safe conclusions have yet to be drawn, as the number of clinical studies is low due to disease rarity and its phenotypic variability (5).

There are very few studies on canine A1ATD, which suggest its connection to chronic liver disease or inflammatory diseases such as panniculitis, polyarthritis, and meningitis in dogs (10, 20–22). This condition is particularly suspected in breeds such as Cocker Spaniels and Bedlington terriers, although it remains unclear whether A1ATD is a direct cause of liver disease or a sign of hepatocellular injury or dysfunction. A case report described decreased A1AT concentrations in a dog with *Bartonella* spp. infection and panniculitis, polyarthritis, and meningitis (22), but another case series could not establish a connection between panniculitis and A1ATD (21). Moreover, the involvement of A1AT (or alpha-1 proteinase inhibitor) has been more thoroughly investigated in canine chronic gastrointestinal disease (23). Alpha-1 proteinase inhibitor (α1-PI) levels in fecal samples can be a helpful diagnostic tool in addition to routine diagnostics and histopathology for diagnosing and monitoring chronic inflammatory gastrointestinal disease (23). Because α1-PI is resistant to breakdown, it is an effective indicator of intestinal protein loss.

To the best of our knowledge, this is the first case of a dog with acinar pulmonary emphysema and pleural effusion associated with A1ATD. The owner reported no exposure to excessive air pollutants or smoking. In our case, no indications suggesting hepatic disease were present. All liver enzymes and liver function parameters were within normal range, and no signs of hepatopathy were detected in ultrasonography or CT. In people with A1ATD, three phenotypes have been reported. In individuals with the lung-predominant phenotype, emphysema can develop independently of liver disease due to the condition’s distinct mechanisms (24). This process is particularly pronounced in individuals with severe deficiency genotypes and is often exacerbated by environmental factors such as smoking or air pollution. In contrast, liver disease in A1ATD arises from the accumulation of misfolded A1AT protein in hepatocytes, a process dependent on specific genetic mutations and protein folding abnormalities (24). While both complications can occur in the same individual, emphysema without liver disease is possible, particularly in cases where protein misfolding and hepatocyte accumulation are minimal, as observed in certain genotypes (7, 24). Spontaneous pneumothorax (SP) represents another clinically significant complication of A1ATD in human patients, typically resulting from the rupture of subpleural bullae in emphysematous lungs. SP may constitute the initial clinical manifestation of previously undiagnosed A1ATD and may recur even after surgical intervention (25). In the current case, CT and thoracotomy did not identify pulmonary bullae, and no episodes of SP occurred during the follow-up period. However, this association remains important for future investigations, as the occurrence of SP in young dogs without trauma or other predisposing factors could indicate underlying A1ATD.

Computed tomography did not reveal any signs of generalized emphysema; however, acinar emphysema was confirmed histopathologically. Similar to findings described in humans (26), round- and lobular-like homogeneous increases in pulmonary parenchymal attenuation that obscured the margins of vessels, airway walls, and air bronchograms, were present on CT. Hypothetically, these changes could correspond to microparticles, such as dust or other environmental contaminants, inducing multifocal pneumonia. Unlike most human cases or other reported dogs with emphysema of different origins (27, 28), our case did not exhibit hyperlucent lung fields, diaphragmatic flattening, or even pulmonary that are responsible for decreased alveolar surface area for gas exchange and contribute to symptoms such as labored breathing, exercise intolerance, and, over time, chronic respiratory failure. Instead, multifocal consolidation (29), rather than emphysema, was primarily observed on CT scan. Moreover, the round lesions noted during the initial CT resolved over time, indicating that a major breakdown of alveolar walls leading to emphysema was not observed during CT, which had a minimal in-plane resolution of 0.4 mm. The resolution of the pulmonary nodules observed on follow-up CT suggests that these lesions were most likely transient inflammatory or infectious foci rather than permanent emphysematous changes attributable to A1ATD. This distinction is important, as emphysema caused by A1ATD is progressive and irreversible, while the disappearance of nodular opacities indicates a separate, self-limiting process. Therefore, the resolution of these lesions does not contradict the diagnosis of A1ATD but rather highlights the concurrent presence of transient pulmonary abnormalities that complicated the diagnostic evaluation. Naturally, it is not possible to differentiate interstitial pneumonia, atelectasis, or tissue breakdown from fluid-filled spaces within consolidated areas by CT. CT consistently underestimates the extent of centriacinar and panacinar emphysema, often missing most lesions less than 0.03–0.04 cm in diameter (30), and is considered insensitive in detecting the earliest or smaller emphysematous lesions in humans (30). This limitation probably applies even more to smaller dogs. However, high-resolution CT was not performed (31). Despite CT’s limited spatial and contrast resolution, histopathology provided significantly higher sensitivity for detecting or confirming acinar emphysema. However, CT showed that the lung was not overinflated.

There are some limitations in our case report. First, the dog was previously treated with multiple antibiotics and glucocorticoids from the referring veterinarian, which could have a noteworthy influence on our diagnosis. Glucocorticoid administration can influence serum A1AT concentrations. As an acute-phase reactant, A1AT hepatic synthesis typically increases in response to inflammation or stress (32). Nevertheless, as the A1AT serum concentration was unmeasurable, an influence of the glucocorticoid treatment on the diagnosis seems unlikely. Furthermore, quantitative A1AT measurement was performed by ELISA and by the validated radioimmunoassay that has been established and could provide more reliable results, as it is independent of antibody specificity (12, 33). The sample yielded a value of 13.5 mg/L, markedly below the published reference interval (RI; >700 mg/L) (12). However, parallel measurement of control serum also resulted in concentrations below the reported RI, suggesting a potential issue with assay performance. To address this, three control serum samples were used for the measurement with radioimmunoassay. Diagnosis of A1AT in people relies not only on quantitative measurement of serum A1AT but also on the detection of the mutation in the SERPINA1 gene. No such analysis is currently accessible for dogs and could therefore not be performed to confirm the gene mutation. Although assay variability and methodological differences can influence results, the concordance of two independent assays, combined with the histopathological finding of emphysema in a young dog without other explanatory causes, provides strong support for a diagnosis of A1ATD. We, therefore, consider the diagnosis to be accurate, while acknowledging that genetic sequencing would represent the gold standard once available in dogs.

Here, we report the first documented case of A1ATD causing persistent pleural effusion and emphysema in a young dog. In our case, the exact pathophysiological mechanism leading to pleural effusion remains uncertain. A plausible explanation is that the lack of A1AT results in increased neutrophil elastase activity and subsequent alveolar wall destruction, which may alter pulmonary microvascular integrity and promote the leakage of low-protein transudate into the pleural space. Additionally, impaired antiprotease defense may contribute to subtle chronic inflammation and increased capillary permeability, even in the absence of overt inflammatory infiltrates. Despite extensive imaging and diagnostic workup, including CT, the emphysematous changes were confirmed only through histopathological examination, emphasizing its superior sensitivity in detecting emphysematous lesions. The absence of hematologic and radiologic evidence of liver involvement is consistent with the lung-predominant phenotype observed in certain genotypes of human A1ATD. Although no specific treatment options are available in dogs, supportive treatment and regular drainage of pleural effusion through a chest port can offer an acceptable quality of life. Further studies are warranted to better understand the clinical presentation, genetic basis, and optimal management of canine A1ATD.

## Data Availability

The original contributions presented in the study are included in the article/supplementary material, further inquiries can be directed to the corresponding author.
